# Region-specific QCT thresholds for osteoporosis assessment in the thoracic spine: why lumbar cut-offs fail

**DOI:** 10.1186/s12891-026-10430-x

**Published:** 2026-09-08

**Authors:** Friederike S. Klockner, Katharina Jäckle, Hassan Awan Malik, Jonathan Roch, Marc-Pascal Meier, Wolfgang Lehmann, Lukas Weiser

**Affiliations:** Klinik für Unfallchirurgie, Orthopädie und Plastische Chirurgie, Universitätsmedizin Göttingen, Göttingen, Germany

## Abstract

**Background:**

Quantitative computed tomography (QCT) is increasingly used for preoperative bone quality assessment in spine surgery. However, established QCT osteoporosis thresholds are based on lumbar reference regions and are frequently extrapolated to the thoracic spine, despite anatomical and biomechanical differences.

**Purpose:**

To evaluate whether lumbar QCT thresholds are transferable to the thoracic spine and to derive provisional thoracic-equivalent thresholds for anatomically defined thoracic subregions by statistical translation of established lumbar American College of Radiology (ACR) thresholds.

**Methods:**

In this retrospective single-center quantitative computed tomography (QCT) study, trabecular volumetric bone mineral density (vBMD, mg/cm^3^) was assessed in 227 patients. Thoracic vBMD was summarized as the mean of Th1–Th10, whereas Th11-Th12 were assigned to the lumbar reference region together with L1-L4 according to the American College of Radiology (ACR) QCT classification scheme. Segmental vBMD distribution was analyzed, and the thoracic–lumbar relationship was modeled using Deming regression. The overall Th1–Th10 conversion was retained as a secondary regional summary; provisional thoracic-equivalent thresholds corresponding to ACR lumbar thresholds (80/120 mg/cm^3^) were derived for upper (Th1–Th4), mid (Th5–Th8), and lower thoracic regions (Th9–Th10).

**Results:**

Thoracic vBMD was significantly higher than lumbar vBMD (102.5 ± 44.1 vs. 85.1 ± 44.6 mg/cm^3^). In patients with evaluable measurements in both regions (*N* = 101), thoracic vBMD exceeded lumbar vBMD by 14.1 ± 19.7 mg/cm^3^ (*p* < 0.001) and showed strong correlation (r = 0.890, *p* < 0.001). A pronounced cranio-caudal decline in vBMD was observed across the thoracic spine (mixed-effects slope − 3.96 mg/cm^3^ per level, *p* < 0.001). Deming regression yielded Th(1–10) = 0.956 × Lum(Th11–L4) + 18.0; the resulting overall Th1–Th10 conversion values of 94 mg/cm^3^ and 133 mg/cm^3^ are reported as a secondary regional summary. Provisional subregional thoracic-equivalent thresholds differed substantially: upper thoracic (Th1–Th4) 118/148 mg/cm^3^, mid thoracic (Th5–Th8) 94/132 mg/cm^3^, and lower thoracic (Th9–Th10) 90/132 mg/cm^3^.

**Conclusion:**

Thoracic vBMD showed a systematic regional offset and substantial cranio-caudal variation. Because the vertebral-level composition of the overall Th1–Th10 mean varied between patients, the overall conversion should not be interpreted as a fixed anatomical thoracic threshold. The anatomically defined subregional values should be regarded as provisional thoracic-equivalent thresholds derived from lumbar ACR criteria and require prospective outcome-based validation before clinical implementation.

## Introduction

Osteoporosis is one of the most prevalent systemic skeletal disorders and represents a major global health burden due to its association with fragility fractures, disability, and increased mortality [1, 2]. In clinical routine, osteoporosis is traditionally diagnosed by bone mineral density (BMD) measurement using dual-energy X-ray absorptiometry (DXA), primarily at the hip and lumbar spine [3]. However, DXA provides only areal BMD and is prone to measurement bias in the presence of degenerative changes, vertebral osteophytes, and vascular calcifications, particularly in older individuals, potentially leading to inaccurate classification of bone status [4, 5].

Quantitative computed tomography (QCT) has emerged as a reliable alternative by enabling three-dimensional volumetric assessment of trabecular bone mineral density (vBMD), which is less affected by degenerative alterations and provides a more direct estimate of vertebral bone quality [6, 7]. In addition, QCT can be applied opportunistically, allowing existing CT examinations obtained for trauma, degenerative spinal disorders, or oncologic staging to be repurposed for bone assessment without additional radiation exposure or patient burden [8]. Given the growing relevance of osteoporosis in spine surgery, accurate regional assessment of vertebral bone quality is increasingly important for preoperative planning, implant selection, and decision-making regarding cement augmentation or alternative fixation strategies.

Currently, ACR practice parameters define diagnostic QCT thresholds for the lumbar reference region, whereas validated thoracic QCT thresholds are not established. In the absence of thoracic-specific reference values, lumbar thresholds are commonly used as the practical comparator in opportunistic spine QCT studies and clinical interpretation, despite relevant anatomical and biomechanical differences between regions. Previous studies have demonstrated that thoracic trabecular bone density is physiologically higher than lumbar vBMD [9, 10], suggesting that direct application of lumbar thresholds may lead to systematic classification differences.

Recent work by Cheneymann et al. proposed thoracic-specific cut-off values derived from linear conversion models, supporting the concept that thoracic bone density requires region-specific interpretation [11]. Compared with the linear conversion model proposed by Cheneymann et al., the present study extends prior work by analyzing a larger cohort, including the upper thoracic spine through Th1-Th10, deriving separate upper, mid and lower thoracic-equivalent values, and applying Deming regression as an errors-in-variables approach rather than ordinary linear regression. This design was chosen because both thoracic and lumbar vBMD measurements are subject to measurement error and because the thoracic spine shows relevant cranio-caudal heterogeneity.

Vertebral bone density varies along the cranio-caudal axis, and the thoracolumbar junction represents a biomechanically unique transition zone with a particularly high incidence of osteoporotic vertebral fractures [12, 13]. Consequently, osteoporosis classification based on a single thoracic reference value may fail to account for relevant segmental differences within the thoracic region.

Therefore, the aim of the present study was to investigate the regional relationship between lumbar and thoracic QCT measurements and to quantify classification discordance when established lumbar QCT thresholds are applied to the thoracic spine. We further sought to derive provisional thoracic-equivalent thresholds for anatomically defined upper (Th1–Th4), mid (Th5–Th8), and lower thoracic (Th9–Th10) subregions by statistically translating the established ACR lumbar thresholds using Deming regression. The overall Th1–Th10 conversion was retained as a secondary regional summary for comparison rather than as a fixed anatomical threshold. We hypothesized that (1) thoracic vBMD is systematically higher than lumbar vBMD despite strong interregional correlation, (2) direct application of lumbar ACR thresholds produces regional classification discordance, and (3) anatomically defined thoracic subregions provide a more appropriate framework for region-specific interpretation than a single thoracic conversion.

## Materials and methods

### Study design and patient selection

This retrospective single-center study included patients who underwent computed tomography (CT) of the thoracolumbar spine between June 2017 and June 2018 at a tertiary university hospital. Inclusion criteria were CT examinations covering both the thoracic and lumbar spine. Examinations with incomplete coverage of the thoracolumbar region were excluded. Patients younger than 20 years were excluded. Fractured vertebrae, vertebrae with osteolysis, severe degenerative destruction, prior instrumentation or major artifacts were excluded on a vertebra-level basis.

A total of 227 patients were included (113 women, 114 men). Mean age was 66 ± 16 years (range 20–92 years). All data were anonymized prior to analysis. The study was approved by the local ethics committee of the University Medical Center Göttingen (approval number 8/11/17). All procedures involving human data were performed in accordance with relevant guidelines and regulations and with the principles of the Declaration of Helsinki. Due to the retrospective and anonymized nature of the analysis, the requirement for informed consent was waived by the ethics committee.

CT indication categories and low-dose protocol status were not prospectively defined as standardized study variables. Retrospective review of the available clinical documentation did not permit a reliable mutually exclusive classification of the indication for the index CT examination in every patient; therefore, indication-specific and low-dose subgroup analyses were not performed. This limitation is considered when interpreting generalizability and potential selection bias.

### CT acquisition protocol

All CT examinations were performed without intravenous contrast on one Siemens SOMATOM Definition AS + scanners (Siemens Healthineers, Erlangen, Germany). Scans were acquired using standardized clinical protocols. Tube voltage was 120 kVp or 80 kVp depending on patient body habitus. Source field of view was 500 mm, reconstruction filters were I30f/B30s and images were reconstructed with a slice thickness of 1.0 mm and an increment of 0.75–1.0 mm using standard reconstruction kernels.

Long-term scanner stability was addressed by regular asynchronous calibration using a Mindways calibration phantom. The calibration phantom was scanned independently on the one CT scanner used for patient examinations at intervals of 4–8 weeks. A numerical scanner-drift analysis was not available for the study period.

### QCT-Based volumetric bone mineral density assessment

Volumetric bone mineral density (vBMD) was assessed using QCT Pro software (Mindways Software Inc., Austin, TX, USA). Trabecular vBMD measurements were obtained using the CliniQCT module, which automatically positioned an elliptical region of interest (ROI) within the trabecular compartment of each vertebral body.

All ROIs were placed using the CliniQCT module and were visually inspected and manually adjusted when necessary to avoid cortical bone, osteophytes, endplate sclerosis, focal lesions or artifacts. Formal intraobserver and interobserver reliability testing of ROI placement was not performed in the original analysis and therefore represents a limitation of this study.

Vertebrae affected by fracture, osteolysis, severe degenerative destruction, prior instrumentation or major imaging artifacts were excluded from analysis. vBMD values were recorded for each evaluable vertebral body from Th1 to L5 and expressed in mg/cm^3^.

### Definition of thoracic and lumbar regions

The primary analysis followed the ACR QCT classification scheme, in which Th11-Th12 were included in the lumbar reference region together with L1-L4. The overall thoracic summary was defined as the mean of all evaluable vertebrae from Th1-Th10. Because scan coverage and vertebra-level exclusions varied between patients, the anatomical level composition contributing to this overall thoracic mean was not identical in every patient. Accordingly, the Th1-Th10 conversion was retained only as a secondary regional summary and was not treated as a fixed anatomical thoracic threshold; region-specific threshold interpretation focused on the anatomically defined subregions.

Because the assignment of Th11-Th12 to the lumbar region is anatomically debatable, a sensitivity analysis was performed in which Th11-Th12 were assigned to the thoracic region and only L1-L4 were used as the lumbar reference.

### Thoracic subregional analysis

To account for physiological variation within the thoracic spine, additional subregional thoracic vBMD values were calculated:

- Upper thoracic spine: Th1–Th4
- Mid thoracic spine: Th5–Th8
- Lower thoracic spine: Th9–Th10

Subregional means were calculated using all evaluable vertebrae within the respective segment. Variable numbers of vertebrae per patient were allowed if at least one evaluable vertebra was available in the subregion.

### DXA Assessment

DXA data were available in 41 patients. DXA examinations performed for medical reasons between June 2017 and December 2018 were evaluated using a GE Lunar iDXA system (GE Healthcare, USA). Areal BMD values (g/cm^2^) and corresponding lumbar DXA T-scores for L1-L4 were extracted from the clinical reports. Patient-level documentation of which individual lumbar vertebrae, if any, had been excluded from the clinical DXA calculation was not systematically abstracted for this retrospective analysis. The exact CT–DXA interval was likewise not systematically abstracted. DXA results were used for descriptive comparison only and were not considered a reference standard for threshold derivation.

### Classification according to ACR QCT thresholds

For comparative purposes, QCT measurements were classified using the ACR practice parameter thresholds for spinal QCT:

- Normal: > 120 mg/cm.^3^
- Osteopenia: 80–120 mg/cm.^3^
- Osteoporosis: < 80 mg/cm.^3^

Although these thresholds were originally defined for the lumbar spine (Th11–L4), they were intentionally applied to thoracic measurements to quantify classification discordance and to evaluate whether lumbar cut-offs are transferable to the thoracic spine.

### Deming regression and derivation of thoracic-equivalent values

For the anatomically defined thoracic subregions, provisional thoracic-equivalent thresholds were derived by statistically translating the established ACR lumbar thresholds (80 mg/cm^3^ and 120 mg/cm^3^). The overall Th1–Th10 conversion was calculated for comparison but was not treated as a fixed anatomical thoracic diagnostic threshold. Deming regression was used as an errors-in-variables approach because both thoracic and lumbar vBMD measurements are subject to measurement error. The variance ratio was set to λ = 1 a priori, corresponding to the assumption of equal measurement-error variances for thoracic and lumbar vBMD. This assumption was considered acceptable because both regional measurements were derived from the same CT examination of each patient, acquired on the same scanner for that examination, using the same QCT software, scanner-specific asynchronous phantom calibration and a standardized ROI workflow.

Deming regression was performed separately for the overall thoracic vBMD summary (Th1–Th10), upper thoracic vBMD (Th1–Th4), mid thoracic vBMD (Th5–Th8) and lower thoracic vBMD (Th9–Th10), each using lumbar reference vBMD (Th11–L4) as the comparator. The Deming regression model was expressed as: Th_region = β₀ + β₁ × Lum_ref, where Th_region represents overall thoracic or thoracic subregional vBMD, Lum_ref represents lumbar reference vBMD, β₀ is the intercept and β₁ is the regression slope. Thoracic-equivalent values corresponding to established lumbar ACR thresholds were calculated by inserting lumbar threshold values of 80 mg/cm^3^ and 120 mg/cm^3^ into the respective regression equations. For overall Th1–Th10 summary, the equation Th1–Th10 = 18.0 + 0.956 × Lum_ref yielded conversion values of approximately 94.5 mg/cm^3^ and 132.7 mg/cm^3^. These were retained as secondary regional summary values rather than fixed diagnostic thresholds.

To evaluate the robustness of the λ = 1 assumption, sensitivity analyses were performed using λ = 0.5 and λ = 2.0. Across these models, the overall Th1–Th10 osteoporosis-equivalent conversion value ranged from 94.2 to 94.7 mg/cm^3^ and the upper/normal equivalent conversion value ranged from 131.4 to 134.0 mg/cm^3^, indicating that the regional conversion was robust. Nevertheless, thoracic measurements may be affected by region-specific artifacts, including rib cage interference, thoracic wall structures and cardiovascular pulsation artifacts, and the assumption of equal error variances remains a methodological simplification.

### Statistical analysis

Statistical analyses were performed using IBM SPSS Statistics (version 25, IBM Corp., Armonk, NY, USA) and additional statistical scripts for Deming regression and bootstrap confidence intervals. Continuous variables are presented as mean ± standard deviation (SD). Normality of continuous variables and paired differences was assessed using Q-Q plots and the Shapiro–Wilk test. Linearity was evaluated by scatter plots. Differences between thoracic and lumbar mean vBMD were assessed using paired t-tests and confirmed using Wilcoxon signed-rank testing where appropriate. Correlations were quantified using Pearson correlation and confirmed using Spearman correlation in sensitivity analysis.

To evaluate segmental variation within the thoracic spine (Th1–Th10), the association between vertebral level and vBMD was assessed using a linear mixed-effects model with vertebral level as a fixed effect and patient as a random intercept to account for repeated measurements within individuals. Confidence intervals for Deming regression parameters and derived thoracic-equivalent values were estimated using nonparametric patient-level bootstrap resampling with 5,000 resamples. Percentile-based 95% confidence intervals were obtained by repeating the complete Deming regression and threshold-translation procedure in each bootstrap resample. Agreement between paired mean thoracic and lumbar vBMD was additionally assessed using Bland–Altman analysis; the mean paired difference and 95% limits of agreement were calculated as the mean difference ± 1.96 standard deviations of the paired differences.

All tests were two-tailed, and a p-value < 0.05 was considered statistically significant.

## Results

### Patient characteristics

A total of 227 patients fulfilled the inclusion criteria and constituted the full retrospective cohort. The mean age was 66 ± 16 years (range 20–92 years); 113 patients were female and 114 male. Complete evaluable paired regional measurements for both mean thoracic vBMD (Th1–Th10) and lumbar reference vBMD (Th11–L4) were available in 101 patients and were used for paired thoracic-lumbar analyses and Deming regression. Patients without complete paired regional measurements were retained for vertebra-level and segmental analyses where applicable, but were not included in complete-case Deming regression. The cohort denominators and analysis sets are summarized in Table 1.

Based on lumbar ACR QCT thresholds in the evaluable lumbar cohort (*n* = 206), 94 patients (45.6%) were classified as osteoporotic, 72 (35.0%) as osteopenic and 40 (19.4%) as normal. Fracture history and comorbidity were not prospectively defined as standardized study variables for the present analysis and were therefore not included in the cohort-level comparison (Table 1).

**Table 1:** Cohort denominators and analysis sets

Analysis set	Definition	N	Use in manuscript
Full retrospective cohort	CT examinations covering thoracic and lumbar spine	227	Patient characteristics, vertebra-level and segmental analyses
Lumbar ACR-evaluable cohort	Patients with evaluable lumbar reference	206	Lumbar ACR prevalence
Complete paired regional cohort	Evaluable mean Th1-Th10 and Th11-L4 values	101	Paired thoracic-lumbar comparison and Deming regression
DXA subgroup	Patients with available lumbar DXA T-Score and lumbar QCT category	41	Descriptive DXA/QCT crosstabulation

The reasons for non-evaluable vertebrae included incomplete scan coverage, fracture, osteolysis, severe degenerative destruction, prior instrumentation and major artifacts. To assess possible selection bias, baseline age, sex and available lumbar vBMD were compared between the paired analysis cohort and the remaining patients. Age (72.2 ± 15.3 vs. 73.3 ± 16.5 years; *p* = 0.606), sex distribution (52 men/49 women vs. 62 men/64 women; *p* = 0.835) and available lumbar vBMD (86.9 ± 42.7 vs. 90.4 ± 43.3 mg/cm^3^; *p* = 0.554) did not differ significantly between groups.

**Fig. 1:**
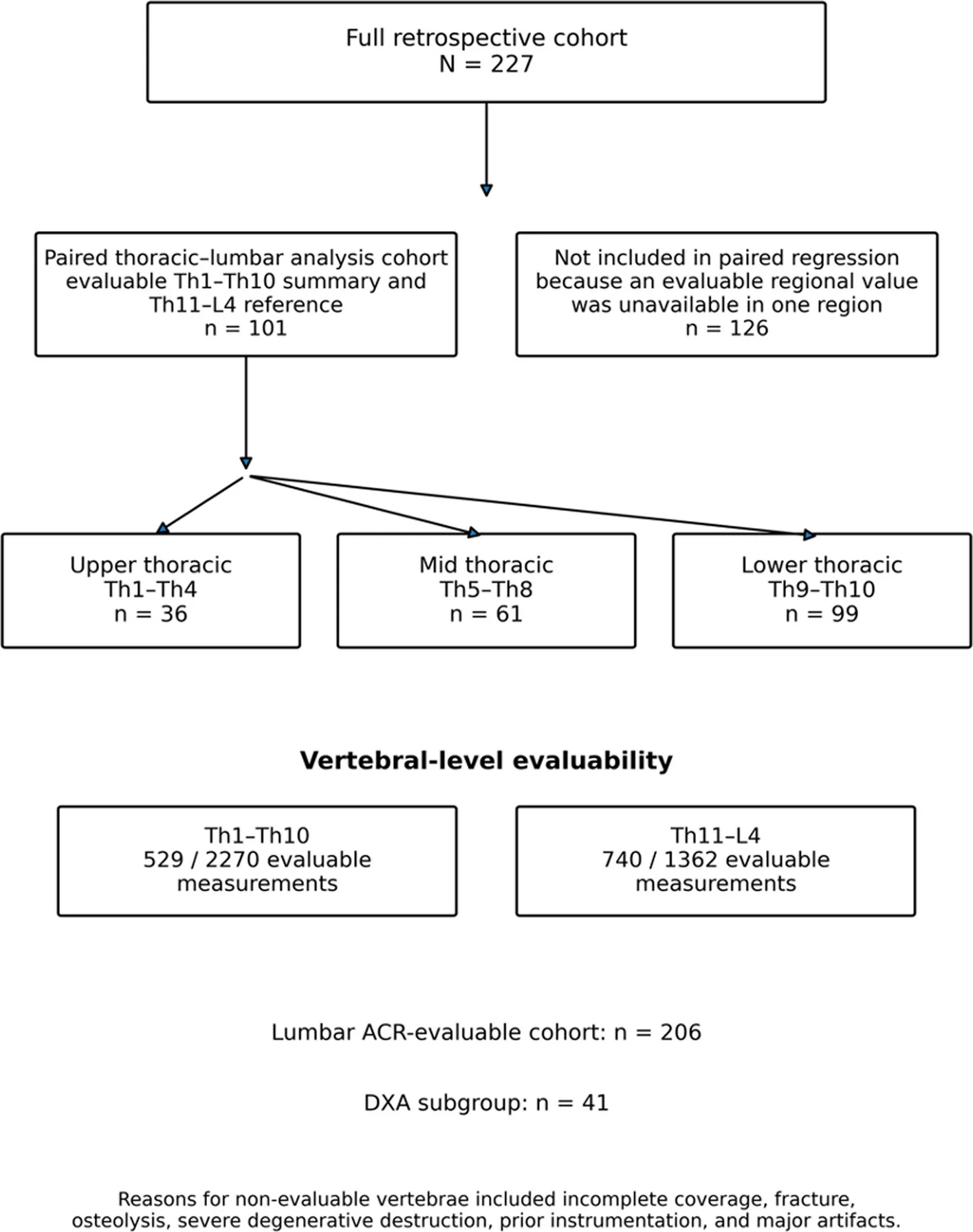
Patient flow and regional evaluability. The figure summarizes the full retrospective cohort, paired thoracic-lumbar analysis cohort, paired subregional analysis sets, vertebral-level evaluability, the lumbar ACR-evaluable cohort and the DXA subgroup. Reasons for non-evaluable vertebrae included incomplete coverage, fracture, osteolysis, severe degenerative destruction, prior instrumentation and major artifacts

At the vertebral level, 529 of 2270 possible measurements from Th1-Th10 and 740 of 1362 possible measurements from Th11-L4 were evaluable. Within the paired regional analysis cohort, the subregional Deming analyses included 36 patients for Th1-Th4, 61 for Th5-Th8 and 99 for Th9-Th10. A mutually exclusive reason-specific count for every non-evaluable vertebral measurement could not be reconstructed because exclusion mechanisms could overlap and were not recorded as a single standardized vertebra-level variable. Patient flow and regional evaluability are summarized in Fig. 1.

### Thoracic vs Lumbar vBMD

Mean volumetric bone mineral density (vBMD) was 102.5 ± 44.1 mg/cm^3^ in the thoracic spine and 85.1 ± 44.6 mg/cm^3^ in the lumbar spine. In patients with evaluable measurements in both regions (*N* = 101), thoracic vBMD values were consistently higher than lumbar values, with a mean paired difference of 14.1 ± 19.7 mg/cm^3^ (*p* < 0.001). Thoracic and lumbar vBMD were strongly correlated (r = 0.890, *p* < 0.001), indicating a stable interregional relationship despite a systematic offset.

**Fig. 2:**
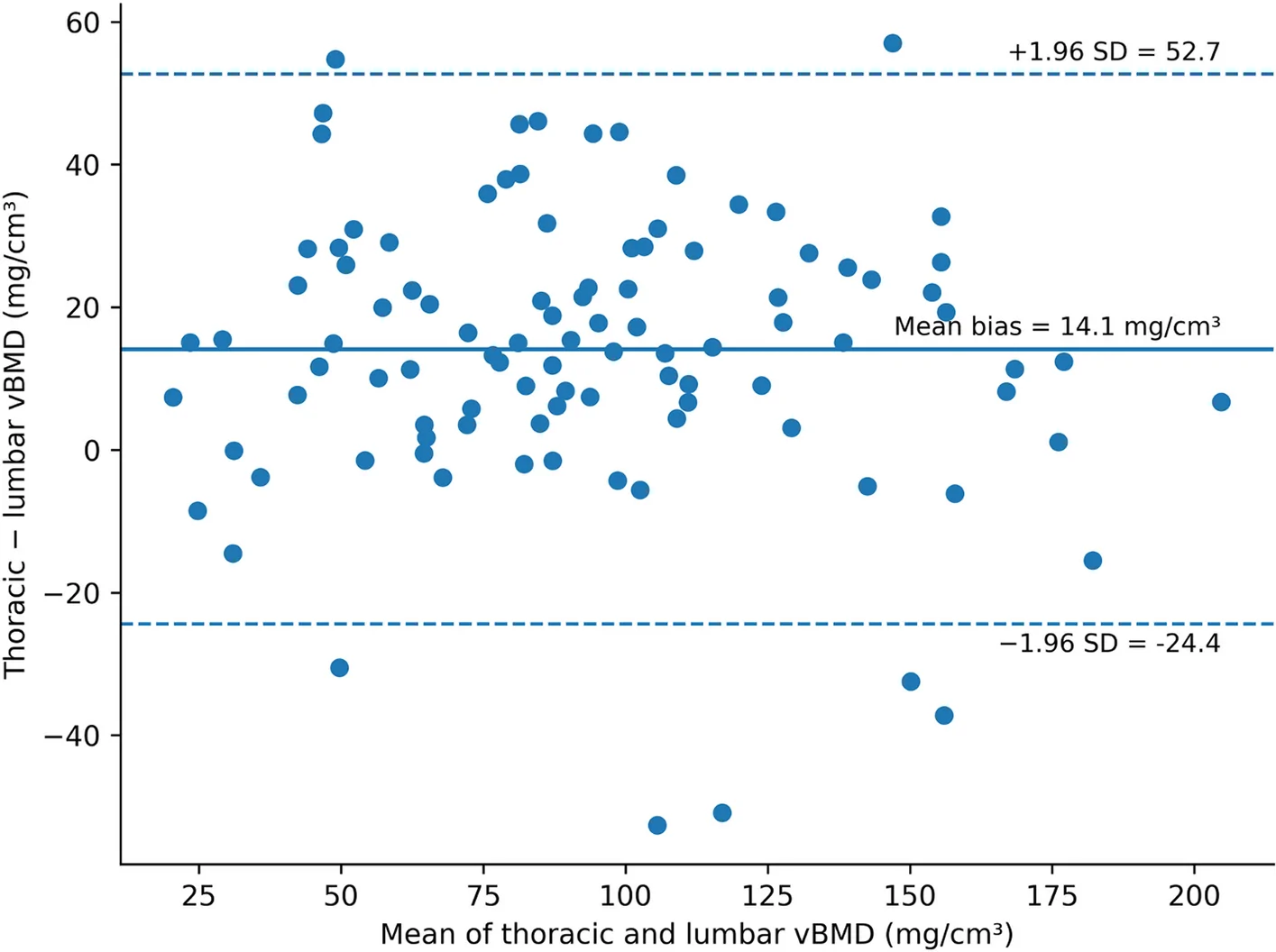
Bland–Altman analysis of paired mean thoracic and lumbar volumetric bone mineral density (vBMD) measurements. The solid line indicates the mean thoracic-lumbar difference (14.1 mg/cm^3^), and the dashed lines indicate the 95% limits of agreement (approximately −24.4 to 52.7 mg/cm.^3^)

Bland–Altman analysis demonstrated a mean thoracic-lumbar difference of 14.1 mg/cm^3^, with 95% limits of agreement of approximately −24.4 to 52.7 mg/cm^3^ (Fig. 2). Thus, the population-level regional offset was clear, but individual thoracic and lumbar values were not interchangeable with sufficient precision for direct patient-level diagnostic conversion.

Segmental analysis demonstrated a cranio-caudal decline in vBMD along the thoracolumbar spine. Mean vBMD was highest in the upper thoracic region and decreased toward the thoracolumbar junction and lumbar spine. When stratified by sex, male patients showed higher vBMD values than female patients across most vertebral levels, while the overall cranio-caudal distribution pattern was comparable between sexes.

### Segmental distribution and thoracic gradient

**Fig. 3:**
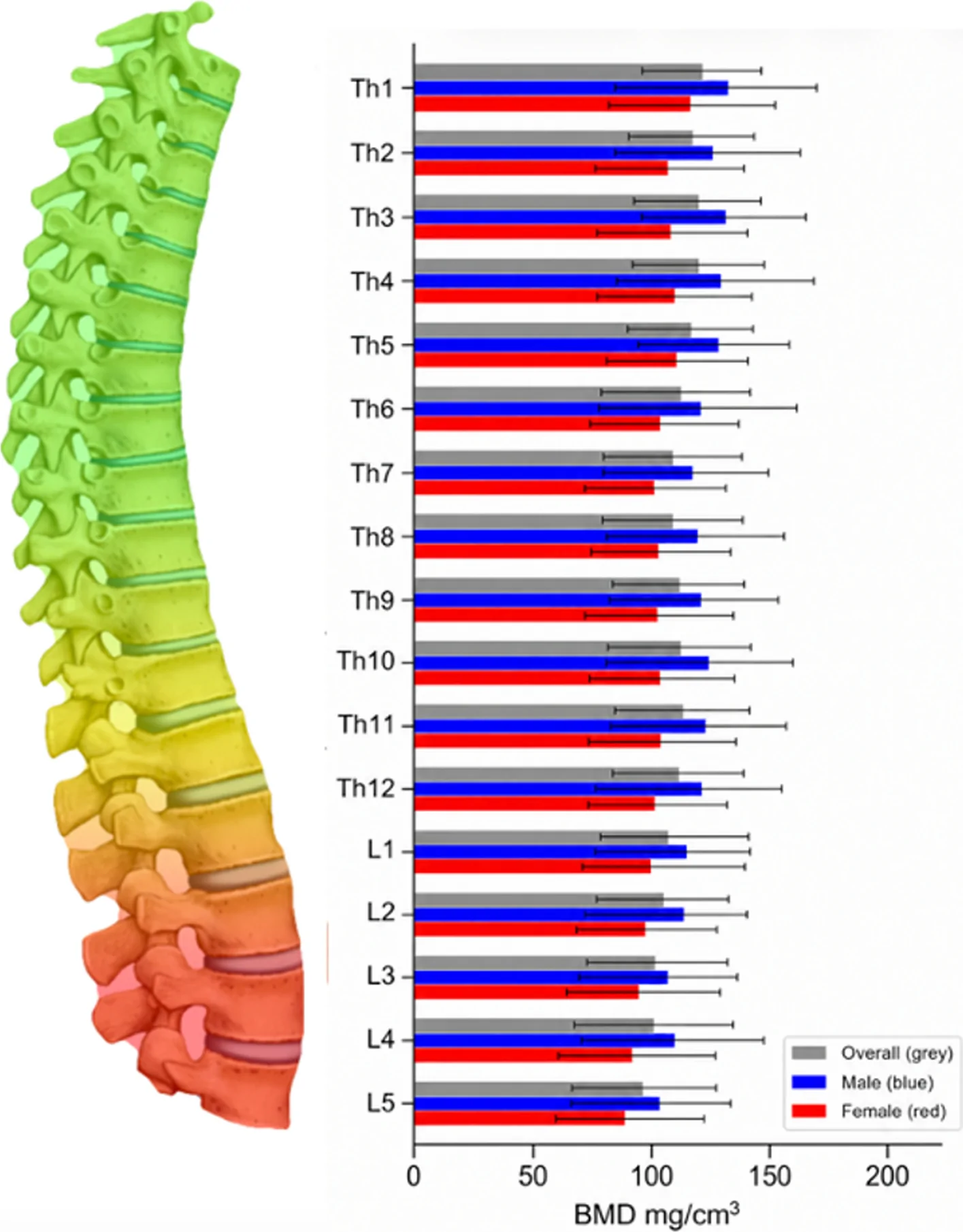
Segmental distribution of volumetric bone mineral density (vBMD) along the thoracolumbar spine. The 3D color-coded rendering illustrates the cranio-caudal vBMD gradient, and the bar chart shows mean vBMD values for each vertebral level from Th1 to L5. Grey bars represent the overall cohort, blue bars represent male patients, and red bars represent female patients. Error bars indicate standard deviation. vBMD was highest in the upper thoracic spine and declined toward the thoracolumbar and lumbar regions

On a segmental level, vBMD demonstrated a pronounced cranio-caudal gradient along the thoracolumbar spine, with highest values in the upper thoracic region and a marked decline toward the thoracolumbar junction and lumbar spine (Fig. 3). Within the thoracic spine (Th1–Th10), vBMD decreased significantly with caudal vertebral level. A linear mixed-effects model (patient as random intercept) confirmed a significant decline of approximately − 3.96 mg/cm^3^ per thoracic level (*p* < 0.001).

Mean vBMD differed between thoracic subregions in the full cohort, with 126.1 ± 42.4 mg/cm^3^ in the upper thoracic spine (Th1-Th4; *n* = 43), 103.5 ± 40.3 mg/cm^3^ in the mid thoracic spine (Th5-Th8; *n* = 71) and 96.4 ± 46.6 mg/cm^3^ in the lower thoracic spine (Th9-Th10; *n* = 111).

### Deming-derived thoracic-equivalent values

Deming regression was performed for the overall Th1–Th10 summary and separately for the upper (Th1–Th4), mid (Th5–Th8), and lower thoracic subregions (Th9–Th10), using lumbar reference vBMD (Th11–L4) as the comparator. For each subregional Deming regression, patients were included when both an evaluable vBMD value for the respective thoracic subregion and a corresponding evaluable lumbar reference vBMD value were available. The Deming regression model was expressed as Th_region = β₀ + β₁ × Lum_ref, where Th_region represents the overall thoracic or thoracic subregional vBMD, Lum_ref represents lumbar reference vBMD, β₀ is the intercept, and β₁ is the regression slope.

For the overall Th1–Th10 summary, Deming regression yielded the relationship:

- Th(1–10) = 0.956 × Lum(Th11–L4) + 18.0 (*N* = 101)

The 95% confidence intervals were 0.859–1.055 for the slope and 9.3–26.8 mg/cm^3^ for the intercept. Based on this model, lumbar ACR thresholds of 80 mg/cm^3^ and 120 mg/cm^3^ corresponded to overall Th1–Th10 conversion values of 94.4 mg/cm^3^ and 132.7 mg/cm^3^, respectively. Because the vertebral-level composition of the Th1–Th10 summary varied between patients, these values are retained as secondary regional summary values and are not proposed as fixed anatomical thoracic diagnostic thresholds.

For the subregional Deming analyses, paired subregional and lumbar reference vBMD values were available in 36 patients for Th1–Th4, 61 patients for Th5–Th8, and 99 patients for Th9–Th10. Subregional analyses demonstrated that the thoracic spine cannot be considered a homogeneous compartment. Deming-based translation of lumbar ACR thresholds resulted in markedly different provisional thoracic-equivalent thresholds for the upper, mid, and lower thoracic spine. In the upper thoracic spine (Th1–Th4), equivalent thresholds were substantially higher (118/148 mg/cm^3^) compared with the mid thoracic spine (Th5–Th8: 94/132 mg/cm^3^) and the lower thoracic spine (Th9–Th10: 90/132 mg/cm^3^). The subregional thresholds and their 95% confidence intervals are summarized in Table 2.

**Table 2:** Provisional thoracic-equivalent thresholds for anatomically defined thoracic subregions, derived by translating the established ACR lumbar reference values (80 and 120 mg/cm^3^; reference region Th11–L4) into thoracic equivalents using Deming regression. Subregional vBMD was evaluable in 43, 71, and 111 patients in the full cohort for Th1–Th4, Th5–Th8, and Th9–Th10, respectively

**Thoracic** **subregion**	**Vertebral levels**	**Provisional thoracic vBMD thresholds** (**mg/cm**^**3**^**)**		
		**Osteoporosis-equivalent threshold**	**Osteopenia-equivalent range**	**Normal-equivalent threshold**
**Upper Thoracic**	Th1-Th4	** < 118 **95% CI: 109.6–126.2	**118–148**	** > 148 **95% CI: 139.4–158.2
**Mid Thoracic**	Th5-Th8	** < 94 **95% CI: 89.2–97.9	**94–132**	** > 132 **95% CI: 125.2–138.3
**Lower Thoracic**	Th9-10	** < 90 **95% CI: 85.7–94.6	**90–132**	** > 132 **95% CI: 123.1–150.8
Lumbar ACR reference*	*Th11-L4*	** < 80**	**80–120**	** > 120**

Deming regression required both an evaluable subregional vBMD value and a corresponding lumbar reference vBMD value, resulting in paired analysis sets of *n* = 36, *n* = 61, and *n* = 99, respectively. Ninety-five percent confidence intervals are shown for the translated threshold estimates. Values are given in mg/cm^3^. Rounded values are provided for clinical interpretability. *Lumbar ACR reference thresholds represent the established lumbar QCT thresholds and were not derived in the present study

### Classification according to ACR thresholds

Applying the original ACR lumbar thresholds directly to mean thoracic vBMD resulted in substantial classification discrepancies. When the full three-category ACR classification comprising normal, osteopenic, and osteoporotic bone density was considered, 28 of 101 patients (27.7%) were assigned to a different category compared with classification based on mean lumbar vBMD. Twelve of these discordant patients had either a thoracic or lumbar regional mean within 10 mg/cm^3^ of the osteoporosis threshold of 80 mg/cm^3^. The discordant subgroup comprised 12 women and 16 men and had a mean age of 71.0 ± 13.0 years.

For the subsequent dichotomized analysis, mean regional vBMD values were classified as osteoporotic (< 80 mg/cm^3^) or non-osteoporotic (≥ 80 mg/cm^3^). Using this threshold, 33 patients were classified as osteoporotic based on mean thoracic vBMD (Th1–Th10), whereas 47 patients were classified as osteoporotic based on mean lumbar vBMD (Th11–L4) (Table 3).

**Table 3:** Discordant osteoporosis classification (< 80 mg/cm^3^) when applying lumbar ACR thresholds to mean thoracic vBMD versus mean lumbar vBMD in patients with complete paired measurements (*N* = 101)

Classification pattern	Thoracic (Th1–Th10)	Lumbar (Th11–L4)	Patients (n)	Proportion (%)
Concordant osteoporotic	< 80	< 80	32	31.7
Concordant non-osteoporotic	≥ 80	≥ 80	53	52.5
Discordant (lumbar osteoporotic only)	≥ 80	< 80	15	14.9
Discordant (thoracic osteoporotic only)	< 80	≥ 80	1	1.0
Total			101	100

When thoracic and lumbar classifications were compared within the same patients using the osteoporosis threshold of 80 mg/cm^3^, 32 patients were classified as osteoporotic in both regions and 53 patients as non-osteoporotic in both regions. A discordant classification pattern was observed in 16 patients (15.8%). In 15 cases (14.9%), lumbar vBMD was classified as osteoporotic (< 80 mg/cm^3^), whereas mean thoracic vBMD was non-osteoporotic (≥ 80 mg/cm^3^). Only one patient (1.0%) showed the inverse constellation, with osteoporotic thoracic vBMD but non-osteoporotic lumbar vBMD.

### Vertebral-level discordance

Vertebral-level discordance was assessed using all evaluable vertebrae within the predefined thoracic (Th1–Th10) and lumbar reference (Th11–L4) regions. Excluded vertebrae did not contribute to the classification, and patients were included in this analysis if at least one evaluable thoracic and one evaluable lumbar reference vertebra were available. On a vertebral level, 41 of 101 patients (40.6%) demonstrated a discordant distribution pattern, defined as at least one thoracic vertebra with vBMD ≥ 80 mg/cm^3^ and at least one lumbar reference vertebra with vBMD < 80 mg/cm^3^. This vertebral-level definition captures within-patient regional heterogeneity and therefore results in higher discordance rates than mean-based regional classification. These patients included 24 women and 17 men and had a mean age of 65.3 ± 9.8 years. Mean thoracic vBMD in this subgroup was 103.4 ± 22.1 mg/cm^3^, whereas the corresponding lumbar reference vBMD was 68.5 ± 14.6 mg/cm^3^ (*p* < 0.001).

### DXA Subgroup

DXA examinations were available in 41 patients and were analyzed descriptively. Based on lumbar DXA T-scores, 13 patients were classified as normal, 16 as osteopenic and 12 as osteoporotic. Cross-tabulation with lumbar QCT categories showed that 27 of 41 patients were classified as osteoporotic by lumbar QCT, 14 as osteopenic and none as normal according to ACR QCT thresholds (Table 4). DXA was not used as the reference standard for deriving thoracic thresholds.

**Table 4:** Descriptive comparison of lumbar DXA and lumbar QCT categories in the DXA subgroup (*n* = 41)

DXA category	QCT osteoporosis (< 80)	QCT osteopenia (80–120)	QCT normal (> 120)	Row total
Osteoporosis	11	1	0	12
Osteopenia	10	6	0	16
Normal	6	7	0	13
Column total	27	14	0	41

## Discussion

The present study demonstrates that thoracic and lumbar volumetric bone mineral density (vBMD) values, although strongly correlated, are separated by a systematic and clinically relevant regional offset. In our cohort of 227 patients, mean thoracic vBMD was 102.5 ± 44.1 mg/cm^3^ compared with 85.1 ± 44.6 mg/cm^3^ in the lumbar spine. In the paired subset with evaluable measurements in both regions (*N* = 101), thoracic vBMD exceeded lumbar vBMD by 14.1 ± 19.7 mg/cm^3^ (*p* < 0.001) despite a very strong interregional correlation (r = 0.890; *p* < 0.001). These findings demonstrate regional measurement correspondence with a systematic offset but do not establish an independent thoracic diagnostic reference standard.

The distinction between correlation and agreement is important for interpretation. Thoracic and lumbar values were obtained from the same patient, CT examination, scanner and calibration workflow, which naturally supports a strong association. Bland–Altman analysis nevertheless showed wide patient-level limits of agreement, indicating that an individual thoracic vBMD value cannot be predicted from the corresponding lumbar value with sufficient precision for direct diagnostic-category conversion. The Deming relationship should therefore be interpreted as regional measurement correspondence rather than proof of thoracic diagnostic validity.

With the aging of the population and the rising incidence of osteoporotic vertebral fractures, preoperative planning prior to spinal instrumentation has become increasingly relevant. In the treatment of unstable osteoporosis-related fractures, trabecular bone mineral density represents a key determinant of fixation strength, as higher trabecular BMD is directly associated with increased pedicle screw anchorage and reduced rates of implant failure in posterior spinal stabilization [14–16]. Consequently, the definition of a reliable threshold for osteoporotic bone quality is essential to enable accurate risk stratification and to guide surgical decision-making.

The consequences of this regional offset become evident when lumbar ACR QCT thresholds are applied to the thoracic spine [17]. Using the ACR cut-off of 80 mg/cm^3^ in the paired subset (*N* = 101), osteoporosis was classified in 47 patients based on mean lumbar vBMD but in only 33 patients based on mean thoracic vBMD. Thus, the same numerical threshold produced different regional classifications, despite the strong correlation between regions. This finding demonstrates classification discordance when a lumbar reference threshold is applied to thoracic measurements,it does not establish which regional classification is clinically correct.

This discordance was particularly pronounced at the vertebral level. On a vertebral level, 41 patients (40.6%) demonstrated a discordant pattern, defined as at least one thoracic vertebra (Th1–Th10) with vBMD ≥ 80 mg/cm^3^ and at least one vertebra within the lumbar reference region (Th11–L4) with vBMD < 80 mg/cm^3^. This vertebral-level definition captures within-patient regional heterogeneity and therefore results in higher discordance rates than mean-based regional classification. These patients included 24 women and 17 men and had a mean age of 65.3 ± 9.8 years. Mean thoracic vBMD in this subgroup was 103.4 ± 22.1 mg/cm^3^, whereas the corresponding lumbar reference vBMD was significantly lower at 68.5 ± 14.6 mg/cm^3^ (*p* < 0.001). This finding suggests that lower thoracolumbar and lumbar segments may more frequently fall below the osteoporosis threshold while thoracic segments remain above it. This is clinically relevant because the thoracolumbar junction represents both a biomechanical transition zone and a common site of osteoporotic vertebral fractures and construct-related complications.

These findings are consistent with prior quantitative imaging studies. Mao et al. reported higher thoracic than lumbar trabecular density in healthy adults [10], and Lenchik et al. demonstrated that thoracic QCT measurements are both technically robust and strongly correlated with lumbar values [18]. More recent opportunistic CT studies by Pickhardt et al. and Graffy et al. further showed that vertebral attenuation varies systematically along the spine, with higher baseline values in the thoracic region [19, 20]. Importantly, QCT precision, reproducibility, and accuracy have been shown to be comparable between thoracic and lumbar vertebrae [21, 22], supporting reproducible regional measurement correspondence. However, the strong same-examination association observed in this cohort does not establish independent diagnostic validity.

From a biomechanical perspective, the observed regional differences are expected. The thoracic spine is integrated into the rib cage and subjected to different loading conditions than the lumbar spine, which serves as the primary weight-bearing segment. Classical biomechanical models have shown that lumbar vertebrae achieve structural stability through larger vertebral bodies and disc geometry, allowing sufficient load bearing at lower trabecular density [12]. Finite element and cadaveric studies further demonstrate that vertebral failure strength depends not only on density but also on geometry and microarchitecture, both of which differ systematically between thoracic and lumbar levels [23, 24]. In this context, the consistently higher thoracic vBMD observed in our cohort likely represents an adaptive response to different load distribution and stabilization requirements rather than a pathological difference.

Importantly, higher thoracic vBMD does not necessarily translate into superior mechanical fixation. Coe et al. showed earlier pedicle screw failure in thoracic compared with lumbar vertebrae despite higher bone density [25]. More recent studies by Schwaiger et al. confirmed that pedicle morphology, cortical thickness, and screw trajectory contribute more to fixation stability than trabecular density alone [26]. These biomechanical considerations reinforce that density-based thresholds must be interpreted in a region-specific context.

From a clinical standpoint, the observed classification discordance may be relevant to preoperative planning, but the present study did not evaluate clinical outcomes. CT- and QCT-based assessment of vertebral bone quality has been associated with pedicle screw fixation strength, screw loosening, vertebral fractures, and construct-related complications [16, 27]. Accordingly, applying lumbar-derived thresholds to thoracic measurements may affect the interpretation of regional bone quality and, in turn, decisions regarding cement augmentation, implant selection, screw trajectory, extension of fixation, perioperative osteoporosis treatment, and postoperative surveillance. However, the derived thoracic-equivalent thresholds in the present study represent statistical translations of established lumbar ACR criteria and have not been validated against fracture risk, treatment decisions, fixation failure, or another clinical reference standard. They should therefore not be used as definitive clinical decision limits.

Our results support the concept proposed by Cheneymann et al. that thoracic bone density requires region-specific interpretation, but differ in several aspects. Cheneymann et al. reported a linear relationship of Thoracic BMD = 0.85 × Lumbar BMD + 33.5, with adjusted thoracic cut-offs of < 102 and > 136 mg/cm^3^. In the present study, Deming regression yielded Th(1–10) = 18.0 + 0.956 × Lum(Th11–L4), resulting in overall Th1–Th10 conversion values of 94.4 and 132.7 mg/cm^3^. In addition, the present study provides provisional thoracic-equivalent thresholds for the upper, mid and lower thoracic spine, demonstrating that a single thoracic conversion may not fully capture cranio-caudal vBMD variation. When lumbar ACR thresholds were directly applied to thoracic measurements, 27.7% of patients were reclassified on a mean-regional basis and 40.6% showed vertebral-level discordance.

The anatomically defined subregional thresholds should therefore be interpreted as statistical translations of established lumbar ACR criteria. They are provisional thoracic-equivalent thresholds, not independently validated thoracic diagnostic cut-offs. The overall Th1–Th10 conversion is retained only as a secondary regional summary because its contributing vertebral levels varied between patients. These results provide testable estimates for future prospective validation against vertebral fracture risk, pedicle screw loosening and construct failure.

The descriptive DXA subgroup further illustrates why the lumbar ACR QCT threshold should be regarded as an anchor for statistical translation rather than an independent clinical gold standard in this cohort. Exact categorical agreement between lumbar DXA and lumbar QCT was 41.5%. Lumbar QCT classified 27 of 41 patients as osteoporotic compared with 12 by DXA; 6 of 13 patients with normal DXA were classified as osteoporotic by QCT, and no patient was classified as QCT-normal. Because DXA and QCT measure different physical quantities and are differently affected by degenerative and structural changes, this discordance should not be interpreted as proof that either method is diagnostically superior. It does, however, show that thoracic-equivalent thresholds translated from lumbar QCT inherit the characteristics and limitations of the lumbar ACR QCT reference classification.

### Limitation

This study has limitations. Its retrospective single-center design may introduce selection bias and may limit generalizability. The provisional thoracic-equivalent thresholds are statistical transformations of established lumbar ACR criteria and have not been independently validated against fracture outcomes, treatment decisions, pedicle screw loosening or construct failure; they should therefore not be interpreted as definitive thoracic diagnostic cut-offs. Thoracic and lumbar measurements were obtained from the same patient, examination, scanner and calibration workflow. This supports measurement correspondence but does not establish diagnostic validity. Thoracic scan coverage and vertebra-level evaluability varied substantially, and because vBMD decreases along the cranio-caudal axis, the overall Th1-Th10 mean did not have identical anatomical composition in every patient. The overall Th1–Th10 conversion is therefore retained only as a secondary regional summary, while threshold interpretation focuses on the anatomically defined subregions. A mutually exclusive reason was not recorded for every non-evaluable vertebral measurement, and exclusion mechanisms could overlap, although aggregate regional evaluability is reported. CT indication categories, low-dose protocol status, examination-level scanner allocation, individual tube-voltage assignments and exact CT-DXA intervals were not systematically abstracted as complete study variables for all patients. Formal interobserver and intraobserver reliability testing of ROI placement was not performed. Finally, the DXA comparison was descriptive only and demonstrated limited categorical agreement with lumbar QCT. Prospective multicenter studies with standardized scan coverage and outcome-based validation are required before the provisional thoracic-equivalent thresholds can be used as clinical decision thresholds.

## Conclusion

In conclusion, direct application of established lumbar ACR thresholds to thoracic QCT measurements produces substantial regional classification differences. Thoracic vBMD was systematically higher than lumbar vBMD and varied substantially by thoracic level. The overall Th1–Th10 conversion should not be interpreted as a fixed anatomical thoracic threshold because its contributing vertebral levels varied between patients. The anatomically defined subregional thresholds should be regarded as provisional thoracic-equivalent translations rather than validated diagnostic cut-offs, and the wide patient-level limits of agreement do not support direct individual conversion from lumbar to thoracic vBMD. Prospective outcome-based validation is required before clinical implementation.

## Data Availability

The datasets generated and/or analyzed during the current study are not publicly available due to institutional data protection regulations and the retrospective nature of the clinical imaging data, but are available from the corresponding author on reasonable request and subject to local institutional and ethical approval.
